# Teachers’ Values as Predictors of Classroom Management Styles: A Relative Weight Analysis

**DOI:** 10.3389/fpsyg.2018.01970

**Published:** 2018-10-16

**Authors:** Daniela Barni, Claudia Russo, Francesca Danioni

**Affiliations:** ^1^Department of Human Sciences, LUMSA University of Rome, Rome, Italy; ^2^Family Studies and Research University Centre, Catholic University of Milan, Milan, Italy

**Keywords:** personal values, socialization values, Schwartz’s theory, classroom management styles, teachers, adolescents

## Abstract

Teachers potentially are important agents of socialization for their students and teachers’ values drive their goals and desirable behaviors. Teachers’ goals and behaviors are also primary influences on students’ achievement motivation and learning. This study – which referred to Schwartz’s Universal Theory of Human Values and involved 157 Italian high school teachers – focused on the relation between teachers’ personal values (i.e., the values teachers feel to be important for themselves) and socialization values (i.e., the values they would like their students to endorse) on the one hand, and their classroom management styles (authoritarian, authoritative, and permissive styles) on the other. Results showed the importance of values in determining the teaching styles, greater in the case of authoritative and authoritarian styles than of permissive style. Implications of these results for teachers’ practices and further expansions of the study are discussed.

## Introduction

Individual values are a topic of great interest in several fields of psychology, such as social, developmental and educational psychology.

Schwartz (1992), in his well-known theory of human values, defined values as *trans*-situational goals that vary of importance and serve as guiding principles in people’s lives. He theorized the existence of ten motivationally distinct value types, which are related to each other into a system. These relations among values can be summarized in terms of a two-dimensional structure composed of four higher-order values. The first dimension contrasts conservation (tradition, conformity, and security), where the emphasis is on self-restraint, preserving traditional practices, and safeguarding stability, and openness to change (hedonism, stimulation, and self-direction), which is instead characterized by the emphasis on change and independence. The second dimension contrasts self-transcendence (universalism and benevolence), where people transcend their selfish concerns to promote the welfare of others, and self-enhancement (power and achievement), where people mainly prioritize their personal interests even at the expense of others.

The available literature has not only analyzed people’s personal values, but also socialization values, namely those values parents and teachers would like their children or students to endorse (Benish-Weisman et al., 2013). Regarding the family, several studies showed that parents act as primary socialization agents for their children (e.g., Fiorilli et al., 2015) and they may differentiate between what is good for themselves (i.e., personal values) and for their children (i.e., socialization values) (Barni et al., 2013). Little is instead known about teachers’ values and their influence onteaching styles, which, as known, have relevant implications for supporting student interest and adjustment and for setting high academic standards (Wentzel, 2002; Bassett et al., 2013). For example, teachers’ personal values have been found to be related to their academic goals and attitudes toward students’ activities (Pudelko and Boon, 2014; Rechter and Sverdlik, 2016). In turn, teachers’ goals and behaviors are primary influences on students’ learning and engagement (Uibu and Kikas, 2014). According to the Self-Determination Theory (Deci and Ryan, 2000), students may be more engaged in school when they develop personal interest toward school activities.

Although psychological research showed personality differences in teaching styles (e.g., Kothari and Pingle, 2015), there are only a few studies on teachers’ personal values and, to our knowledge, none of them empirically treated the existing relations between teachers’ personal and socialization values.

## The Present Study

Referring to Schwartz’s (1992) theory of values, we measured the importance high-school teachers gave to conservation, openness to change, self-transcendence, and self-enhancement, both in terms of personal (i.e., important for one’s self) and socialization values (i.e., important to transmit to one’s students).

The study explored the relationships between these values and teachers’ classroom management styles, namely the degree and type of involvement and control of teachers with their students. Applying the framework of parenting style to the classroom (Bassett et al., 2013), we distinguished three teaching styles: authoritative, authoritarian and permissive. The authoritative teacher exhibits a warm and nurturing attitude toward his/her students and expresses interest and affection, thus placing limits and controls on the students while simultaneously encouraging their independence. The authoritarian teacher, who focuses on discipline and has expectations of swift obedience, believes that the students only need to pay attention during classes to gain knowledge. Finally, the permissive teacher is not engaged with his/her students or learning, places few demands on the students, and does not try to manage the classroom environment.

Using a relatively new analytic strategy, that is the relative weight analysis (Johnson, 2000), we analyzed the relative importance of each value in the context of the others, addressing these questions: “How much can teachers’ values explain their classroom management style?” “Which values (personal vs. socialization) are the most important predictors of teachers’ classroom management styles?” In answering these questions, we controlled for teachers’ gender and number of years of teaching (e.g., Martin et al., 2006; Çelebi, 2014).

## Method

### Participants and Procedure

Participants were 157 Italian teachers (68.9% females), working in public (83.1%) or private (16.9%) high schools of Northern Italy, aged between 25 and 67 years (*M* = 44.1, *SD* = 9.46). On average, teachers had been working in the schools for 8.05 years (*SD* = 7.91, range = 1–34).

Participants were recruited with the collaboration of the schools. Teachers who gave written informed consent filled out an anonymous self-report questionnaire; they were also told that there was no right or wrong answer. The study was approved by the Scientific Committee of the Family Studies and Research University Centre, Catholic University of Milan, and followed the APA ethical guidelines of research.

### Measures

#### Personal Values

The 21-item Portrait Values Questionnaire (PVQ; Schwartz, 2003) was used. The PVQ is composed of 21 verbal portraits of a person and his/her objectives or aspirations, which indirectly reflect the importance of a value. For example, “Thinking up new ideas and being creative is important to him/her. He/She likes to do things in his/her own original way” describes a person for whom openness to change is important. Respondents’ values are inferred from their self-reported similarity (from 1 = not like me at all to 6 = very much like me) to people described. We computed four mean indexes assessing the importance personally given to conservation (α = 0.67), openness to change (α = 0.69), self-transcendence (α = 0.62), and self-enhancement (α = 0.77).

#### Socialization Values

The PVQ was once again used. For each of the 21 portraits, teachers answered on a 6-point Likert scale (1 = I would not want it at all to 6 = I would want it very much) the extent to which they wanted their students to be like the person described. We computed four scores for teachers’ socialization values, namely conservation (α = 0.75), openness to change (α = 0.68), self-transcendence (α = 0.79), and self-enhancement (α = 0.78).

#### Teachers’ Classroom Management Styles

Three subscales (i.e., authoritative, authoritarian, permissive) from the Classroom Management Profile (Kearney, 2008) were used, for a total of 9 items, and respondents were asked to answer on a 5-point Likert scale (1 = strongly disagree to 5 = strongly agree). An item example is: “I am concerned about both what my students learn and how they learn” (authoritative style). We computed three mean indexes assessing the authoritative (α = 0.60), authoritarian (α = 0.64), and permissive teaching styles (α = 0.59).

### Data Analysis

#### Preliminary Analysis

We described the study variables in terms of means, standard deviations and range. Associations between variables were measured by bivariate Pearson correlations.

#### Relations Between Teachers’ Personal and Socialization Values and Classroom Management Styles

To investigate whether and how teachers’ personal and socialization values predicted their classroom management styles, we performed three multiple linear regressions (MR) and relative weight analyses (RWA), controlling for teachers’ gender (contrast-code -0.5 = male, 0.5 female) and years of teaching.

Throughout MR, we estimated the overall R^2^ and determined the statistical significance of individual regression coefficients (i.e., unique contribution). However, when predictors are correlated – as likely in the case of the teachers’ personal values and socialization values – MR is not enough to adequately divide variance in the criterion among the predictors (Barni, 2015). We therefore combined MR with RWA, which uses a variable transformation approach to address the issue of correlated predictors and focuses on the proportionate contribution each predictor makes to R^2^, considering both its unique relation with the criterion and its relation when combined with other predictors (i.e., relative contribution) (Johnson, 2000).

## Results

Descriptive statistics of the study variables and their correlations are reported in **Table 1**, which showed strong correlations between teachers’ personal and socialization values. Teachers gave the greatest importance to self-transcendence, followed by openness to change, conservation and self-enhancement. The same value hierarchy was found for teachers’ socialization values.

**Table 1:** Pearson correlation coefficients, means, standard deviations and ranges of teachers’ values and classroom management styles (*N* = 157).

	1	2	3	4	5	6	7	8	9	10	11
*Personal values*											
(1) Conservation	1	0.10	0.30^∗∗^	0.24^∗∗^	0.77^∗∗^	0.05	0.13	0.23^∗^	-0.01	0.28^∗∗^	-0.06
(2) Openness to Change		1	0.20^∗^	0.52^∗∗^	0.18^∗^	0.62^∗∗^	0.19^∗^	0.44^∗∗^	0.19^∗^	0.18^∗^	0.01
(3) Self-transcendence			1	-0.02	0.19^∗^	0.19^∗^	0.63^∗∗^	-0.01	0.30^∗∗^	-0.03	0.10
(4) Self-enhancement				1	0.34^∗∗^	0.44^∗∗^	-0.07	0.75^∗∗^	-0.09^∗^	0.26^∗∗^	-0.08
*Socialization values*											
(5) Conservation					1	0.25^∗∗^	0.18^∗^	0.40^∗∗^	-0.06	0.29^∗∗^	-0.03
(6) Openness to Change						1	0.27^∗∗^	0.51^∗∗^	0.14	0.20^∗^	0.06
(7) Self-transcendence							1	-0.08	0.26^∗∗^	-0.01	0.12
(8) Self-enhancement								1	-0.17^∗^	0.22^∗∗^	0.03
*Classroom management styles*											
(9) Authoritative style									1	0.04	-0.06
(10) Authoritarian style										1	-0.15
(11) Permissive style											1
Mean	3.74	3.75	4.83	2.75	3.67	3.73	5.22	2.50	4.40	2.90	2.94
SD	0.78	0.75	0.63	0.96	0.85	0.70	0.65	0.86	0.46	0.63	0.61
Range	1.85–5.62	2.30–5.90	2.80–6.00	1.00–5.43	1.67–6.00	2.33–6.00	3.25–6.00	1.00–5.25	3.00–5.00	1.33–5.00	1.33–4.33


*^∗∗^*p* < 0.01, ^∗^*p* < 0.05.*

With regard to authoritative and authoritarian styles, the MR predictors, respectively, yielded a *R*^2^ = 0.19 and *R*^2^ = 0.15. As the inspection of β coefficients suggests, teachers’ personal values were stronger associated with these teaching styles compared to teachers’ socialization values. The more teachers personally recognized the importance of openness to change and self-transcendence, the more they were prone to adopt an authoritative style; the less important was self-enhancement, the more they were authoritative. The more teachers perceived conservation values as important principles in their lives, the more they endorsed an authoritarian style.

Teachers’ values overall explained a small percentage of the variance for permissive style (*R*^2^ = 0.05). Indeed, neither personal nor socialization values were significantly related to this teaching style. Similarly, teachers’ gender and years of teaching were not significant predictors of any classroom management style, except for gender in predicting the authoritative style. Specifically, female teachers reported being more authoritative than male teachers.

Interestingly, likely due to the high correlations between personal and socialization values (**Table 1**), the RWA results were quite different from those of MR. RWA reevaluated the contribution of socialization values, which all together accounted for 47.2% of the explained variance of authoritative style and for 40.4% of authoritarian style explained variance (**Table 2**).

**Table 2:** The importance of teachers’ personal and socialization values in predicting classroom management styles.

	Authoritative style (*R*^2^ = 0.19)				Authoritarian style (*R*^2^ = 0.15)				Permissive style (*R*^2^ = 0.05)			
	MR		RWA		MR		RWA		MR		RWA	
	β	*p*	Raw importance	Rescaled importance	β	*p*	Raw importance	Rescaled importance	β	*p*	Raw importance	Rescaled importance
*Personal values*												
Conservation	-0.07	0.407	0.01	0.1%	0.33	0.000	0.05	31.2%	-0.07	0.458	0.00	6.1%
Openness to Change	0.29	0.004	0.03	15.7%	0.13	0.198	0.01	7.1%	0.01	0.945	0.00	2.6%
Self-transcendence	0.23	0.014	0.05	26.3%	-0.17	0.074	0.01	4.8%	0.14	0.173	0.01	13.2%
Self-enhancement	-0.22	0.028	0.01	5.3%	0.11	0.282	0.02	15.2%	-0.06	0.566	0.02	25.0%
*Socialization values*												
Conservation	-0.00	0.993	0.01	5.3%	0.10	0.513	0.03	20.2%	-0.10	0.502	0.00	3.5%
Openness to Change	0.01	0.963	0.02	10.5%	0.17	0.170	0.02	12.3%	0.02	0.891	0.00	4.7%
Self-transcendence	0.10	0.396	0.03	15.7%	-0.06	0.598	0.01	1.1%	0.17	0.170	0.01	19.5%
Self-enhancement	-0.28	0.039	0.03	15.7%	-0.13	0.347	0.00	6.8%	0.25	0.088	0.01	17.5%
Gender	0.18	0.041	0.00	5.3%	-0.09	0.332	0.00	1.2%	-0.05	0.565	0.00	7.6%
Years of teaching	0.06	0.477	0.00	0.1%	0.00	0.994	0.00	0.1%	-0.02	0.860	0.00	0.3%
			0.19	100%			0.15	100%			0.05	100%


*Rescaled importance (%) was computed by dividing the relative weights by the total *R^2^* and multiplying by 100.*

## Discussion

This study focused on high-school teachers’ personal values (i.e., the values teachers feel important for themselves) and socialization values (i.e., the values teachers would like their students to endorse) and showed these values were related to teachers’ classroom management style.

The combined use of MR and RWA allowed us to disentangle the specific contribution of each value in explaining teachers’ classroom management style (i.e., authoritative, authoritarian, and permissive styles), taking into account the motivational (and statistical) interdependence among values. Teachers’ classroom management style was found to be shaped not only by teachers’ personal values, but also by those values teachers wanted their students to endorse. Interestingly, authoritative style, which is characterized by warmth and control and is unanimously considered the most beneficial for students (Buskist and Benassi, 2012), was the most dependent on teachers’ personal values as well as on socialization values. The more teachers gave great importance to self-transcendence and openness to change and little importance to self-enhancement for themselves and in the relationship with their students, the more they adopted an authoritative teaching style. Authoritarian style, which is characterized by high standards and expectations of obedience, was instead slightly more related to teachers’ personal values, especially to conservation, than to socialization ones. Teachers who felt tradition, conformity, and security as important values for their life, showed to prefer an authoritarian teaching style. Interestingly, these results are consistent with Pudelko and Boon’s (2014) study concerning teachers’ values and their classroom goals, which showed that self-transcendence and openness to change were associated to mastery and prosocial approaches, while conservation encouraged performance and compliance goals. Our findings suggest that authoritative style is guided by a complex pattern of values and is a more other-oriented style (i.e., “what could be important for my students”) than the authoritarian one, which is instead more self-focused (i.e., “what is important for myself”) and guided mainly by one value, namely conservation.

Finally, permissive teaching style was weakly related with teachers’ values. This style is in some respects a form of disengagement from the situation (e.g., teachers place very few demands from students and do not care much about how students are doing) and research concerning the value-behavior link showed that values predict behaviors only in situations that are construed in relevant terms (Vallacher and Wegner, 1987). This result, however, should be interpreted with caution given the low reliability of permissive style score and needs to be deepened in future studies, also including contextual factors (e.g., school climate). Further shortcomings of this study include the involvement of a single-country convenience sample and its cross-sectional design.

The strongest point of the study is instead to have provided further insight into teacher personal characteristics that are related to general classroom management style. It showed that values, both personal and socialization ones, can help improve teachers’ skills in managing behaviors and classroom setting and create a positive learning environment. That is, self-knowledge and conscious endorsement of personal values as well as of values to be transmitted may be central to quality teaching. A positive quality of teaching in fact does not only depend on teachers’ ability to transmit knowledge but also something “valuable,” that might become a resource in their students’ lives.

## Data Availability

The datasets for this manuscript are not publicly available because of local legal and privacy restrictions (Italian Data Protection Code – Legislative Decree No. 196/2003).

## Ethics Statement

The authors assert that all procedures contributing to this work comply with the ethical standards of the relevant national and institutional committees on human experimentation. All participants gave written informed consent in accordance with the Declaration of Helsinki. The study was approved by the Scientific Committee of the Family Studies and Research University Centre, Catholic University of Milan, Italy.

## Author Contributions

DB designed and carried out the study, contributed to the analysis of the results, and wrote the manuscript. CR and FD contributed to the analysis of the results and wrote the manuscript.

## Conflict of Interest Statement

The authors declare that the research was conducted in the absence of any commercial or financial relationships that could be construed as a potential conflict of interest. The reviewer CF declared a shared affiliation, with no collaboration, with several of the authors, DB and CR, to the handling editor at time of review.
